# Ovary abortion is prevalent in diverse maize inbred lines and is under genetic control

**DOI:** 10.1038/s41598-018-31216-9

**Published:** 2018-08-29

**Authors:** Jeffery L. Gustin, Susan K. Boehlein, Janine R. Shaw, Weschester Junior, A. Mark Settles, Ashley Webster, William F. Tracy, L. Curtis Hannah

**Affiliations:** 1 Program in Plant Molecular and Cellular Biology, Genetics Institute and Department of Horticultural Sciences, University of Florida, Gainesville, FL 32611 USA; 2 Florida Agricultural and Mechanical University, Tallahassee, FL 32301 USA; 3 Department of Agronomy, University of Wisconsin, Madison, WI 53706 USA

## Abstract

Crop improvement programs focus on characteristics that are important for plant productivity. Typically genes underlying these traits are identified and stacked to create improved cultivars. Hence, identification of valuable traits for plant productivity is critical for plant improvement. Here we describe an important characteristic for maize productivity. Despite the fact mature maize ears are typically covered with kernels, we find that only a fraction of ovaries give rise to mature kernels. Non-developed ovaries degenerate while neighboring fertilized ovaries produce kernels that fill the ear. Abortion occurs throughout the ear, not just at the tip. We show that the fraction of aborted ovaries/kernels is genetically controlled and varies widely among maize lines, and low abortion genotypes are rare. Reducing or eliminating ovary abortion could substantially increase yield, making this characteristic a new target for selection in maize improvement programs.

## Introduction

A grand challenge to our society is feeding the growing human population while facing unfavorable climate change. Plant improvement programs are tasked with increasing yield on less land, in harsher environments, and with reduced inputs for plant growth. A myriad of plant characteristics ranging from disease, pest, and abiotic stress resistance, to photosynthesis, respiration, and nitrogen fixation, are targets for plant improvement. The kernel or grain, the harvested unit for many crops, has also received particular attention. Maize, or corn, is the largest yielding crop in the US with over 380 million metric tons produced in 2016^1^. The maize kernel is a staple food in the diet of much of the world’s population, is a major feed source for livestock production, and provides raw materials for many products that we use in our daily lives.

Grain yield in maize is a product of individual kernel weight and kernel number; hence, factors affecting them have received substantial attention^2–6^. The potential number of kernels is determined by the number of mature florets on the ear inflorescence. Each floret contains an ovary with a single megametophyte that can develop into a kernel^7^. Failure of kernels to develop from ovaries, here termed ovary abortion reduces grain number on the ear. Kernel number, rather than kernel weight, has the larger impact on yield^8^. Therefore, strategies that maximize kernel number could translate into increased yield.

The typical maize ear is covered with kernels, and their removal reveals no evidence of undeveloped ovaries or kernels. Hence, it is surprising, as documented here, that only 60 to 65% of the maize ovaries commonly give rise to a functional, developed kernel under typical field conditions. We show that genetic variation exists for ovary abortion, hence making this trait potentially amendable to genetic selection for improved yield in maize breeding programs.

## Results

### Ovary abortion is common in maize inbred lines

**Figure 1:**
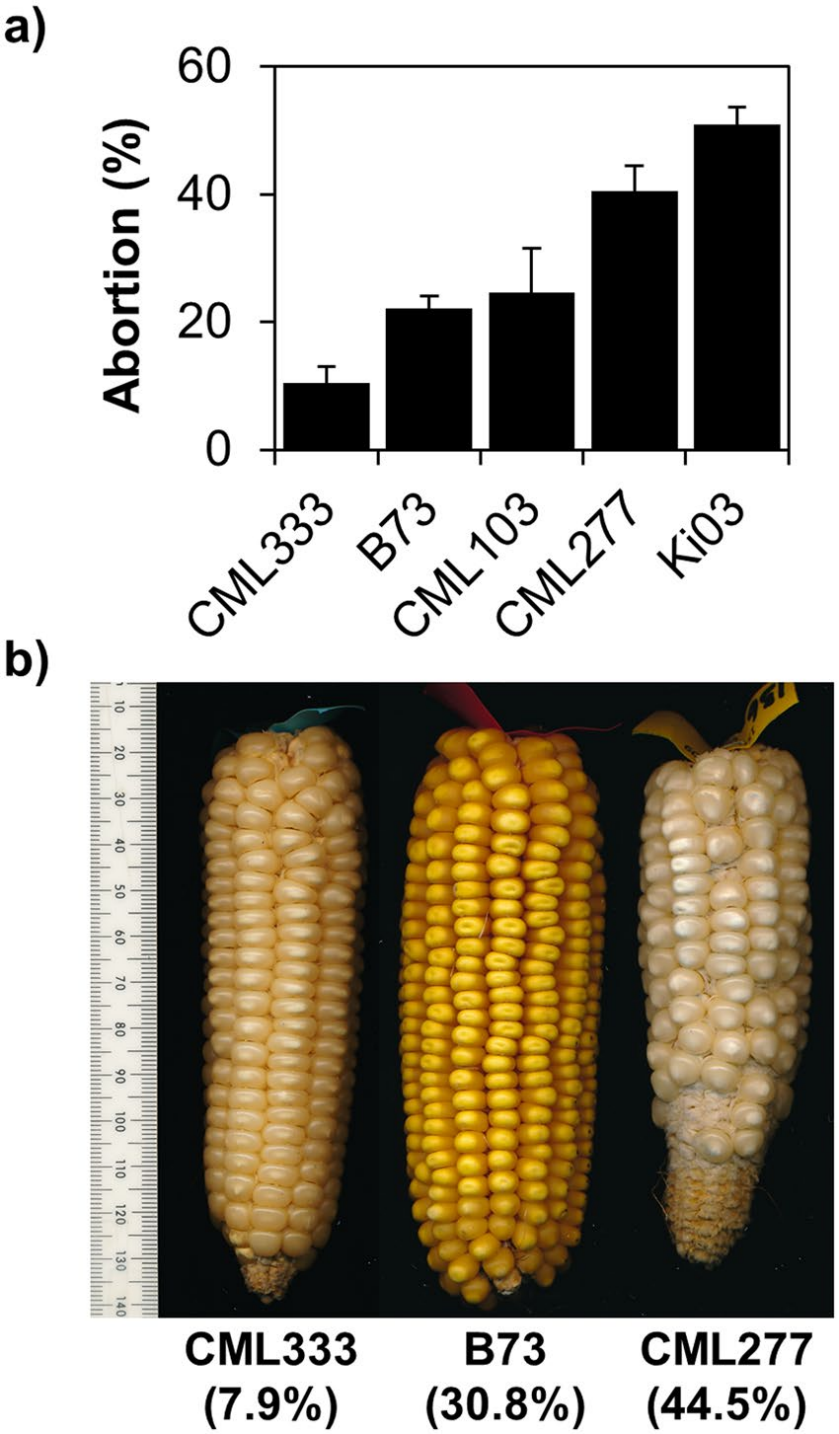
Ovary abortion varies among diverse inbred lines. Selected inbred abortion rates (**a**) and selected ears (**b**) from 2015 fall nursery with low, average, or high abortion phenotypes. The numbers in parentheses are the percentages of missing kernels on the imaged ears. Error bars represent standard error of the mean.

Prompted from observations of poor kernel set in experimental maize lines^9^, we counted the number of kernels that failed to develop on ears of the 26 parental inbred lines of the maize Nested Association Mapping (NAM) population^10^. These lines contain tropical, temperate, sweet corn and popcorn types and represent a majority of the common genetic variation found in maize^11^. The number of kernels that failed to develop was determined by comparing the number of silks 4–5 days after their emergence to the number of developed kernels at maturity. Plotted in Fig. 1a is the percentage of silks that do not give rise to a developed kernel in five inbred lines spanning the phenotypic spectrum. Abortion among the diverse inbred lines ranged from less than 10% to over 80% (Supplementary Table S1). Many ears visually identified as having full kernel set actually had relatively large rates of abortion (Fig. 1b). For example, the imaged B73 ear lost more than 180 kernels (31%), yet appears to have similar kernel set as the CML333 ear which lost less than 10% of its kernels.

**Figure 2:**
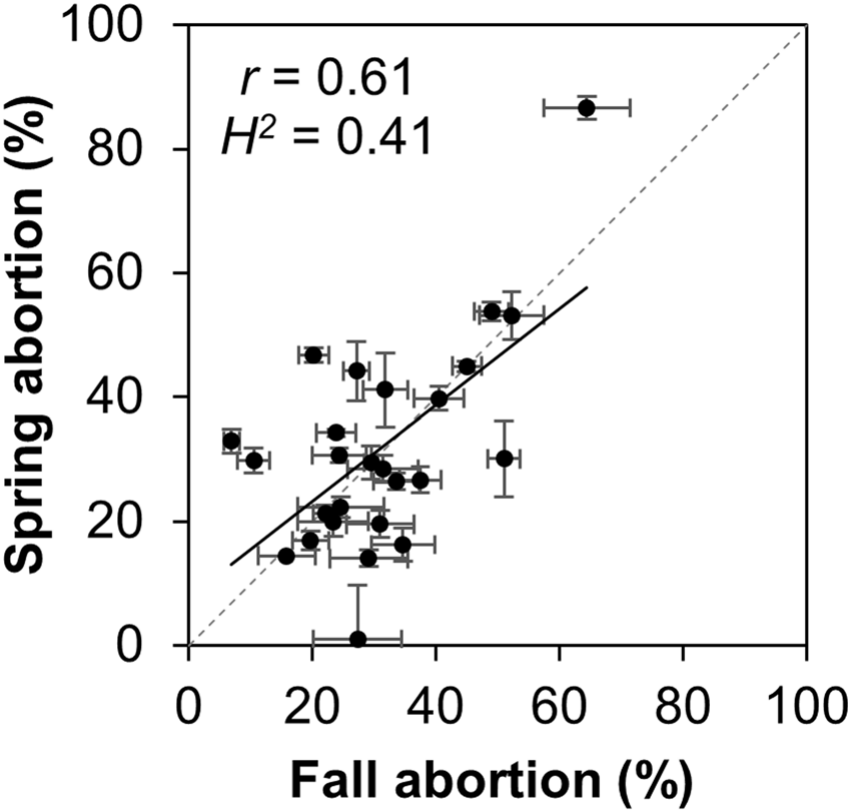
Ovary abortion among diverse maize inbred lines. The scatterplot shows mean values from 26 NAM parental inbreds grown in 2015 spring and fall nurseries. The plot shows a linear regression trend line (solid line) and one-to-one correspondence line (dashed line). Error bars represent standard error of the mean calculated from 6 ears per data point on average. Correlation coefficient (*r*) and broad sense heritability (*H*^*2*^) are shown.

Importantly, abortion was significantly correlated (r = 0.52, p < 0.001) and heritable (H^2^ = 0.62) across multiple seasons (Fig. 2). Plants grown in the Florida spring nursery (April-July) are typically exposed to increasing day length and temperature during the growing season, in contrast to the fall season (August-December) when cooler temperatures promote slower plant and kernel development. Despite these different growing conditions, there was no difference in mean abortion between the two seasons (*p* = 0.42, Welch two-sample *t*-test). Average abortion based on silk and mature kernel counts in the spring crop was 33% ± 14% standard deviation and 30% ± 15% standard deviation in the fall crop. While the materials analyzed here were inbred maize lines, it is interesting to note that commercial hybrids have also shown substantial kernel loss. A Mycogen hybrid exhibited 43% kernel loss under non-stressed growing conditions^9^, two Nidera hybrids grown under optimal field conditions lost more than 30% of kernels^12^, and a Dekalb hybrid varied between 32 and 38% kernel loss in unstressed treatments^13^. These observations suggest that sensitivity to kernel loss in some hybrids is on par with the mean kernel loss in the inbred lines used in this study.

### Ovary abortion occurs around the time of pollination and is distributed throughout the ear

**Figure 3:**
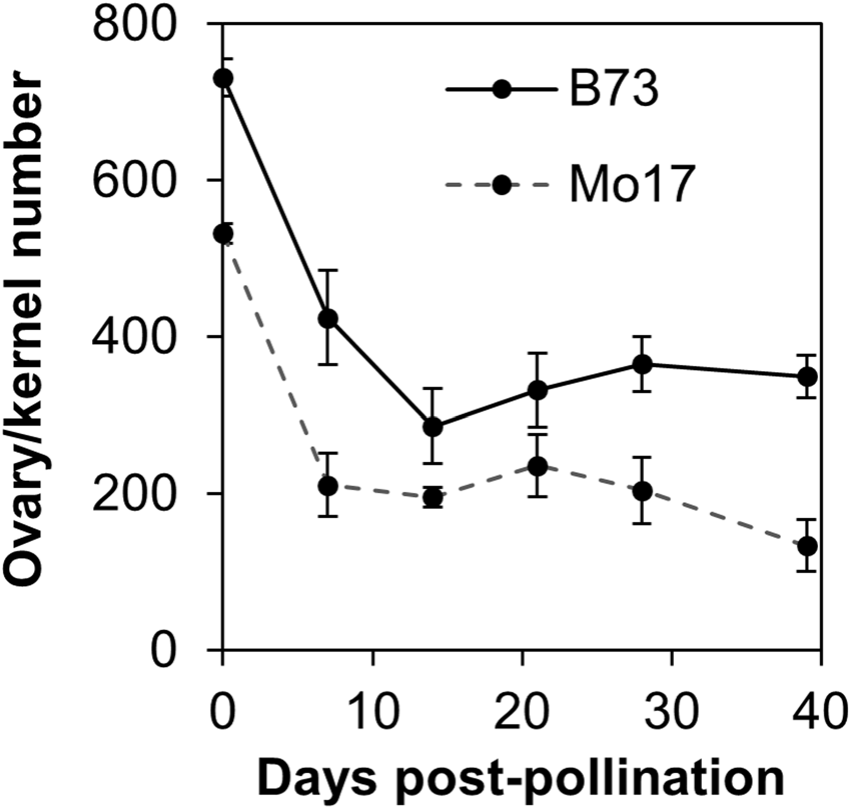
Enlarging ovaries or kernels as a function of development in inbreds B73 and Mo17. Shown at 0 days post-pollination (DPP) is the average number of ovaries on an unpollinated ear. Shown at 40 DPP is the average number of developed kernels on a mature ear at harvest. Error bars represent standard error of the mean calculated from 5 ears per data point on average.

Failure to form a kernel could occur for a variety of reasons, such as a defective ovary, pollination failure, or abortion of the fertilized ovary. In this study, the terms innate ovary abortion and kernel loss are used for all these scenarios, although failure of a fertilized ovary to develop into a fully mature kernel would be kernel rather than ovary abortion. To define the timing of kernel failure, we compared the number of ovaries on non-pollinated ears, enlarging ovaries during development, and final number of developed kernels in two maize inbred lines, B73 and Mo17. These lines were chosen because of their historic importance in maize improvement programs and because they differ in final kernel number. While B73 ears contained 731 ± 53 ovaries before pollination, mature ears exhibited only 362 ± 61 kernels at maturity (Fig. 3). The loss of 50.5% of potential kernels in B73 occurred early in development since only 400 ± 143 ovaries exhibited any enlargement seven days post-pollination. Analogous data were obtained from the inbred Mo17. Initial ovary number averaged 532 ± 28 while mature ears contained only 134 ± 65 (25.2%) kernels. Like B73, a substantial number of the initial ovaries exhibited no enlargement, since only 240 ± 23 (45.1%) increased in size seven days after pollination. In contrast to B73, not all enlarged ovaries at seven days post-pollination developed into kernels. Despite the apparent late stage loss in Mo17, the majority of potential kernels in both inbred lines were lost before or soon after pollination.

**Figure 4:**
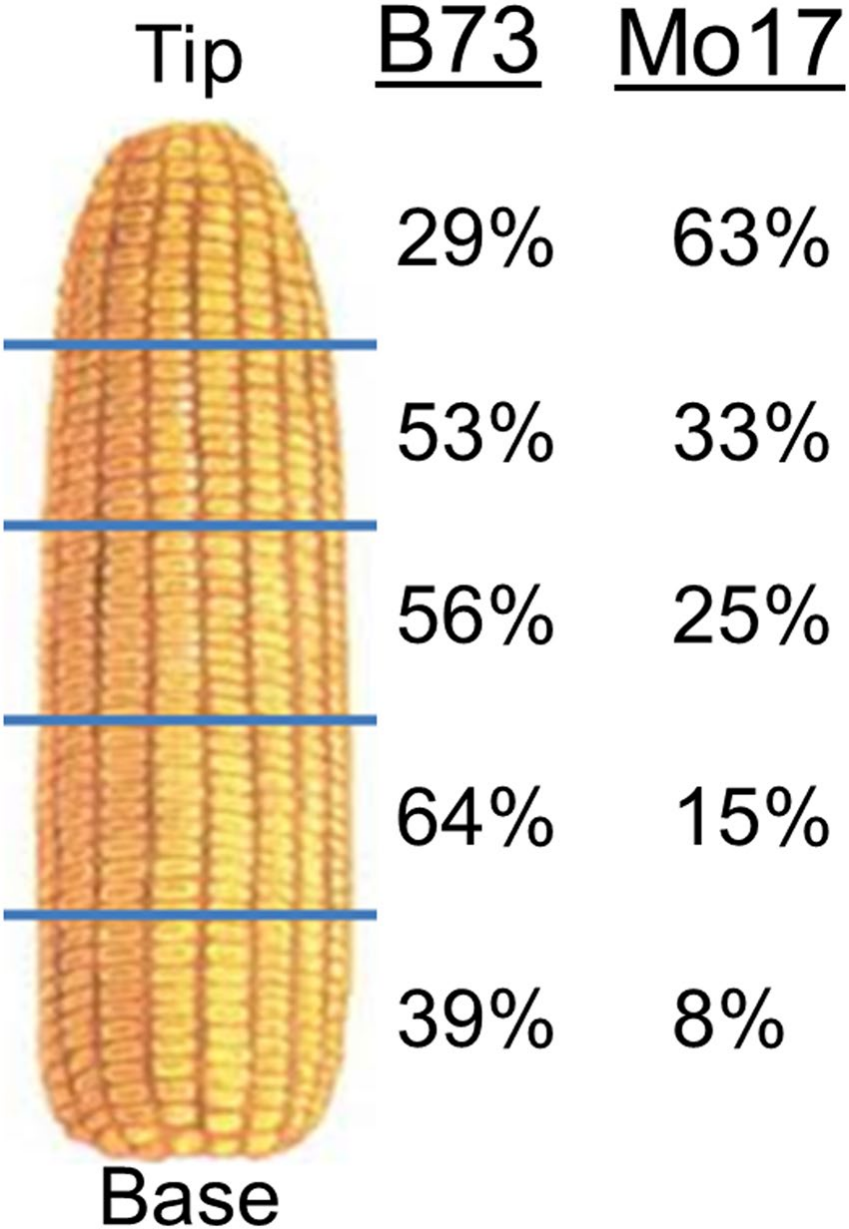
The percentage of ovaries giving rise to developed kernels at five locations along the ear in B73 and Mo17 inbred lines.

**Figure 5:**
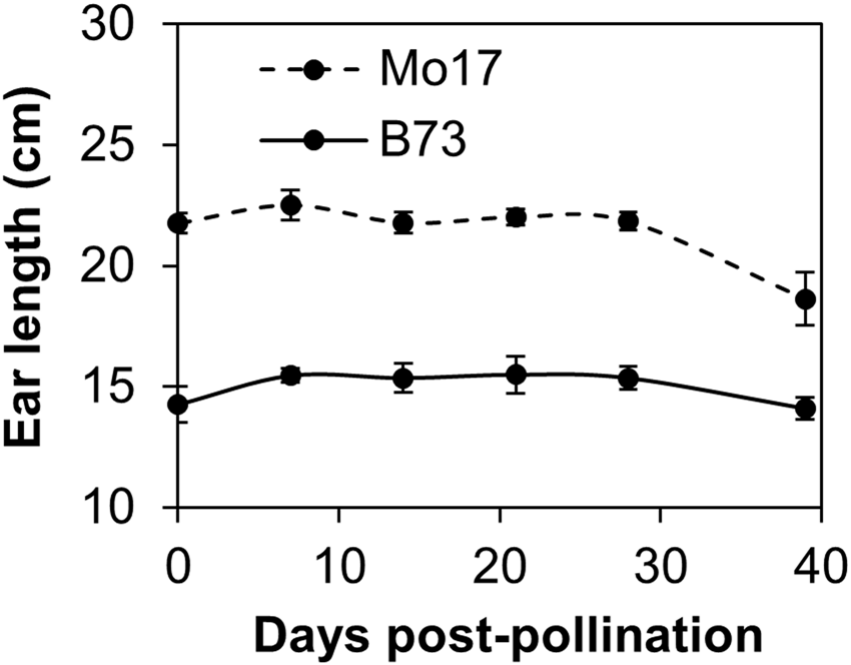
Ear length as a function of development. Error bars represent standard error of the mean.

In the maize female inflorescence, florets at the base of the ear mature earlier than those at the tip and are first to draw upon the photoassimlate supply^14^. These developmental and proximity gradients have implications for which ovaries will set seed when resources are limiting^14^. For example, drought stress at pollination induces kernel loss primarily at the tip of the ear^15,16^. To test whether innate abortion is asymmetrically distributed, ears of B73 and Mo17 were divided into five equal sections and the numbers of ovaries, enlarging ovaries, and mature kernels were determined during ear development. The percentages of ovaries giving rise to a developed kernel in the five regions of the ear are shown in Fig. 4. The highest frequency of kernel development occurred in the midsection of B73 ears, with reduced kernel set at the extremities of the ear. Mo17 exhibited an entirely different pattern. The highest percentage of kernel development occurred at the tip of the ear, while the lowest probability for kernel development occurred at the base. Unlike drought stress, the probability of kernel development exhibited a gradient on the ear and was genotype specific. Importantly, cob length following silk emergence of B73 and Mo17 changed only slightly (Fig. 5), showing that ovary position remained stationary during development. This ensured that the frequency of kernel development at various locations on the ear could be determined.

Because kernel loss occurs at the time of pollination or soon after, the number of silks was counted to determine whether inadequate silk number caused kernel losses. The number of silks equaled 84% and 91% of the number of ovaries in B73 and Mo17, respectively (Table 1). A sweet corn inbred containing the *shrunken-*2 mutation was also evaluated to expand this data set. Ovary number equaled 517 ± 109, whereas 487 ± 93 silks were observed. Silk number in all three lines was comparable to ovary number; hence the number of silks was not the primary factor limiting kernel set^12,17^.

**Table 1:** Number of ovaries and silks two days post-silking on non-pollinated ears.

Genotype	No. silks	No. ovaries	percentage
B73	648 ± 51	775 ± 60	84
Mo17	470 ± 44	514 ± 31	91

Values are means and standard errors calculated from five ears per data point on average.

Temperatures at the Florida nursery are typically hot during kernel set in the summer season. The average daily high and low temperatures during the first 12 to 15 days following fertilization of the B73 and Mo17 genotypes were 33.6 °C and 21.9 °C, respectively (http://fawn.ifas.ufl.edu/). Accordingly, the experiment was repeated in the more moderate climate of Madison, Wisconsin during the unusually mild 2014 growing season where the average high and low daily temperature during pollination were 26 °C and 15 °C, respectively. In Wisconsin, kernel set for Mo17 and B73 equaled 46% and 45% of ovaries, respectively (Table 2). While kernel set for Mo17 was higher in Wisconsin than in Florida, the lower temperatures of Wisconsin did not produce kernel set ratios approaching 100%.

**Table 2:** Number of silks, ovaries and developed kernels in maize grown in Madison, Wisconsin.

Genotype	No. Silks	No. Ovaries	No. Kernels
B73	577 ± 50	738 ± 71 (^a^78%)	331 ± 30 (^b^44.8%)
Mo17	452 ± 66	519 ± 68 (^a^87%)	241 ± 93 (^b^46%)

The numbers in parentheses are the percent of ovaries that produce ^a^silks or ^b^kernels. Values are means and standard errors calculated from 5 ears per data point on average.

The silk and ovary data show that the silk counts used to measure kernel loss in Fig. 1 and Supplementary Table S1 most likely underestimate the number of ovaries and therefore underestimate kernel loss. Incorporation of this adjustment leads to the conclusion that only 61.4% of the ovaries give rise to a developed kernel, on average, in the NAM inbred lines.

### Kernel development accompanies disintegration of neighboring ovaries

**Figure 6:**
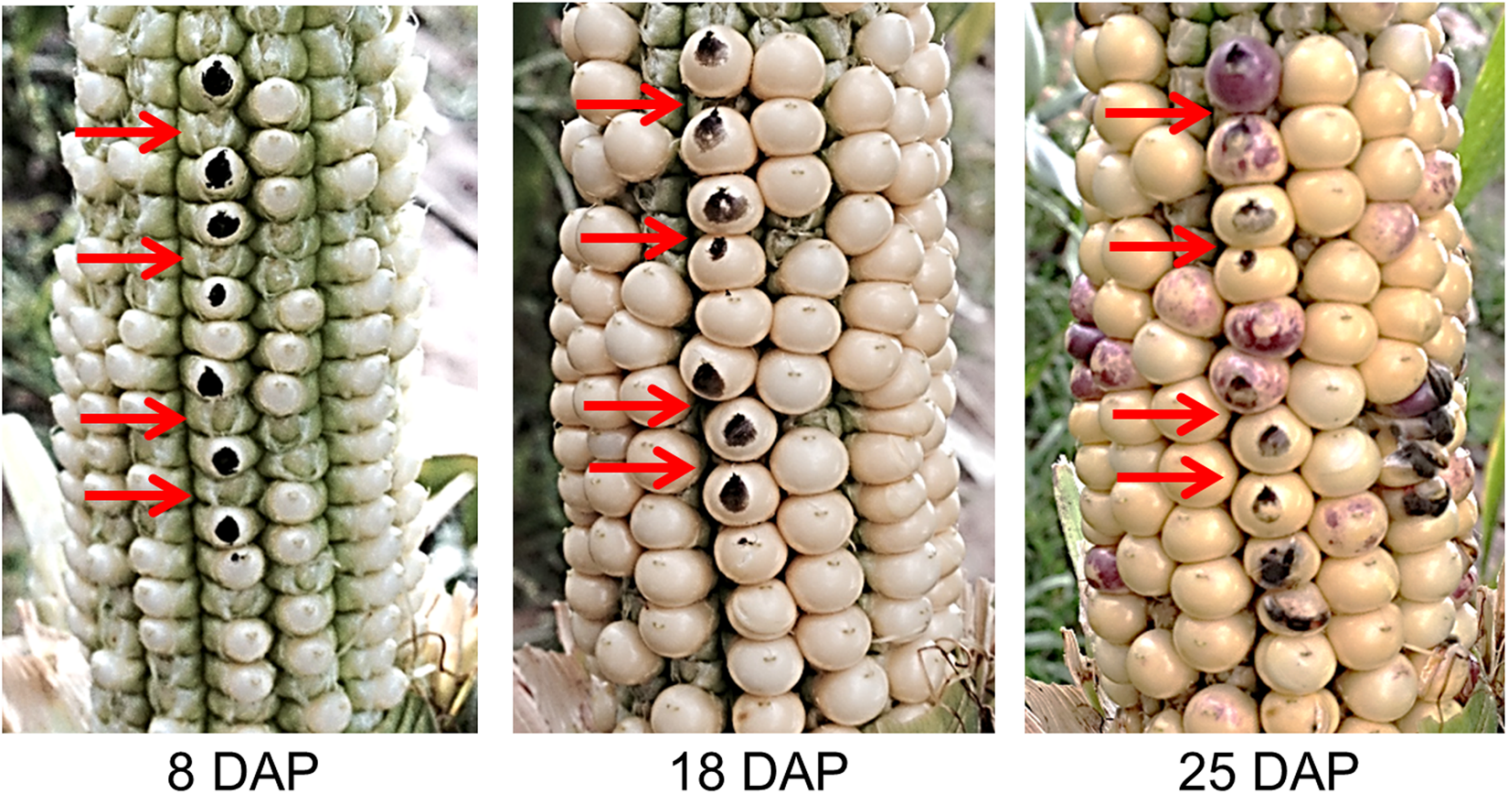
Disappearance of space occupied by non-developing ovaries. Sequential images of a single developing W22 inbred ear after hand pollination. The ear was husked 8 days post-pollination (DPP). Red arrows point to four selected ovaries that did not develop into kernels.

Examination of typical maize ears revealed no ovaries remained on the cob following kernel removal. To gain further insight into the disappearance of ovaries during development, we measured the number of ovaries on non-pollinated ears at two days post-silking and at the time of normal ear maturity (40 days post-pollination in the Florida spring environment). Non-pollinated Mo17 ears contained 460.5 ovaries at two days post-silking and 411.5 ovaries (89.4%) at maturity. Corresponding ovary counts for non-pollinated B73 were 730.5 and 680.3 (93.1%). Hence in the absence of fertilization and kernel development, ovaries remain intact and do not disintegrate during the maturation of the plant. However, upon fertilization the non-developing ovaries and the space they occupy are consumed by expansion of neighboring kernels. Time-lapse images of a W22 inbred ear illustrate how space of undeveloped ovaries is filled during development (Fig. 6). Ovaries not enlarging can be distinguished on the ear eight days post-pollination (DPP). The space occupied by the non-developing ovaries at eight DPP is covered by developing kernels during the next 17 days of development. The lack of visual evidence of ovary abortion on the mature ear shows that the degree of kernel loss cannot be determined by examining the ear at harvest (Fig. 1b).

Taken together, these data show that only 60 to 65% of maize ovaries in diverse inbred lines commonly develop into mature kernels. Ovaries not fertilized, or those that are fertilized but do not develop, are consumed as nearby kernels develop. How the “develop/do not develop” decision is made is not understood. It is interesting to note that mutant maize kernels lacking the gene expression necessary for early kernel development, termed empty pericarp, are not selected against in this process since the pericarp develops and is present in the mature ear. Many of the defective kernel mutants exhibit typical Mendelian 3 to 1 ratios in F_2_ generations^18–22^, suggesting that post-fertilization seed developmental defects are not a factor in the abortion decision.

## Discussion

We found that ovary abortion is significant in maize grown in conventional field conditions, and genetic variation exists for this trait. Hence, maize breeders now have an additional trait to exploit in maize breeding programs. The extent to which reduced ovary abortion and how it is impacted by stress conditions will affect yield is presently unknown. However, it is noteworthy that expression of trehalose phosphate phosphatase (TPP) in developing maize florets increased kernel number and increased yield in hybrids under non-stressed conditions^23^. Furthermore, maize transgenes encoding an enzyme in the starch biosynthetic pathway reduced ovary abortion, and thereby increased kernel number without decreasing individual kernel weight^9,24^. These data suggest that mitigating ovary abortion, even in a hybrid context^9^, can positively impact yield under optimal cultivation conditions.

The cellular mechanisms underlying ovary abortion are not understood. Many previous studies of ovary abortion in maize have involved the imposition of an environmental stress at the time of kernel set, when maize is most susceptible to kernel loss. Drought stress imposed three days prior to silking is sufficient to substantially reduce kernel set^25,26^. Failure to set kernels is not due to lack of fertilization, but rather abortion of the floral ovary near the time of pollination. Two explanations for this abortion have been put forward.

One hypothesis focuses on altered carbohydrate metabolism^2,27,28^. Starch reserves in ovaries are depleted after several days of drought stress when senescence-related transcripts begin to accumulate marking the onset of abortion^4,5^. Addition of exogenous sucrose to water-stressed plants partially restores kernel set^2^, suggesting that carbohydrate status of the ovary is critical for determining whether an ovary will set seed during environmentally stressful conditions.

Consistent with the hypothesis that inadequate carbohydrate supply reduces kernel set, the sugar trehalose-6-phosphate (T6P) has been implicated in ovary abortion in maize. T6P is a metabolic intermediate of the trehalose biosynthetic pathway and is an important signaling molecule that integrates sugar status with growth and development in plants^29–32^. T6P is produced by trehalose phosphate synthase (TPS) from UDP-glucose and glucose-6-phosphate, and T6P is converted to trehalose via trehalose-6-phosphate phosphatase (TPP)^33^. The T6P concentration closely follows that of sucrose in most tissues. However, in floral tissues T6P and sucrose levels were found to be inversely related around the time of pollination, with T6P levels increasing as sucrose levels decrease^34^. Decoupling of the T6P/sucrose relationship in floral tissues may be important for seed set. Nuccio *et al*.^23^ found that reducing T6P by transgenically expressing TPP in maize florets increased sucrose content and significantly increased kernel number. The increase in kernel number was most likely due to reduced ovary abortion, although the ovary number was not counted. The slight increase in sucrose in floral organs was proposed to mitigate ovary abortion and increased kernel number in drought stressed plants.

In an alternative hypothesis, Oury *et al*.^17,35^, invoked drought-induced reductions in cell expansion and silk growth as the major cause of ovary abortion. These investigators noted that low light intensities caused the same reduction in the amount of photosynthate entering the ovary as caused by water stress, yet severe abortion did not occur. They found that water stress delayed silk emergence and the time period of total silk growth. Under extreme drought, some silks did not emerge from the husks and hence could not be used for fertilization. Furthermore, expression analysis showed that genes involved in cell wall properties were among the first to be altered, rather than genes involved in carbohydrate metabolism. Hence, these observations point to lack of silk growth as the cause of reduced kernel set. Oury *et al*.^17,35^, do note that their data do not exclude the possibility that carbohydrate status may also play a role, so the two models are not mutually exclusive. Genetic variants, as noted here, should aid in identifying the actual mechanism of drought induced seed abortion.

While studies of stress-induced ovary abortion invariably find that most abortion occurs at the tip of maize ears^14,17^, it is surprising that the abortion described here under typical field conditions does not follow this pattern. In this case, abortion occurs throughout the ear. While abortion in B73 is greatest at the tip, a substantial number of kernels are also lost at the middle and base of the ear. The pattern in Mo17 is striking. The highest seed set is actually at the tip of the ear, with a gradient of reduced seed set toward the base of the ear. Hence, abortion can occur at any location along the ear, and does not always proceed basipetally. This observation has bearing on the mechanism of abortion. It is unclear why one floret would fail, while neighboring florets proceed to kernel development. Currently proposed mechanisms, based upon basipetal patterns of abortion, predict that neighboring ovaries have different ovary/silk growth rates^17,35,36^ or carbohydrate levels^28,32,34,36^. However in multiple hybrid lines, neighboring ovaries were found to have very similar silk growth rates and carbohydrate contents^17,35^. It is possible that florets can suppress the development of their immediate neighbors through well-established dominance signals^37^. But it should be noted that pollinations of the NAM inbred lines were conducted synchronously, such that all available silks received pollen at once. This should have ensured synchronous fertilization of the ovaries and avoided the confounding effects of early fertilized kernels preventing the development of kernels on later silking florets as found in natural pollinations^36^.

In summary, our observations reveal a little reported, but potentially important phenotype, innate ovary abortion, which could have important implications for maize breeding. A substantial number of ovaries do not develop under typical field conditions, and there is genetic variation for this trait. Hence, ovary abortion is a viable target phenotype for breeding maize hybrids with higher seed set. This study reveals patterns of abortion not noted in previous studies and identifies genetic variation can be used to decipher the mechanisms of kernel abortion.

## Materials and Methods

Plants were grown at the University of Florida Plant Science Research and Education Center in Citra, Florida, or at the University of Wisconsin farm facility near Madison, Wisconsin. Plots in Florida were sown with a low planting density of approximately 30,000 plants ha^−1^ using 36 inch row spacing. Fertilizer was applied four times during the growth period, totaling 240 lbs N, 54 lbs P and 225 lbs K per acre. Water was provided as needed by overhead irrigation. Plots were kept well-watered. Each inbred was grown in a single block surrounded by other corn lines to maximize pollination. Pollinations within each block of B73 and Mo17 inbred lines were synchronized^6^ such that pollination initiation of experimental ears within each inbred occurred on the same day (June 13, 2013 for B73 and June 17, 2013 for Mo17). Only plants having silks visible under the coverings for one to two days were chosen for pollination. Coverings were removed and ears were hand pollinated with pollen from sibling plants. Ears remained uncovered to allow subsequent pollinations from neighboring plants. Ears were harvested at 0, 7, 14, 21 and 28 days post-pollination (DPP). Ears were also harvested at maturity (around 40 days in the Florida spring environment). Each developmental time point was represented by at least five ears. Ear length was measured and each ear was divided into five equally-sized sections. The number of enlarging and non-enlarging ovaries, as well as the number of developed kernels on mature ears, was determined in each section. Ovary number on non-pollinated ears harvested at the normal time of maturity was also determined. Silk number on ears harvested at 0 DPP was also determined. Non-pollinated ears harvested at approximately two days post-silking as well as pollinated ears at maturity were harvested in Wisconsin and shipped to Florida for counting. All data were obtained by manual counting.

The 26 NAM inbred lines have a wide range of flowering times. To minimize the influence of pollination date on measured traits, the lines were planted at staggered dates according to their flowering times and all pollinations were conducted within a 17 day and 13 day window in the 2015 spring and fall nurseries, respectively. Pollinations for the NAM parental lines were conducted as described for B73 and Mo17 above except that ears were cut back to the ear tip one day prior to pollination and the ears were covered after sibling pollinations preventing pollination from neighboring plants. Ovary number was estimated from the number of silks counted at the time of pollination (Table 1) and therefore slightly underestimate the total number of ovaries on the ear (Tables 1 and 2).

### Statistical Analysis

Broad-sense heritability (*H*^2^) was defined as: *H*^2^ = MSgen (MSgen + MSyear + MSer + MSgenMSer)^−1^, where MSgen is the genotype mean square, MSyear is the season mean square, and MSer is the error mean square from a two-way ANOVA.

## Electronic supplementary material

Dataset 1

## Data Availability

The datasets generated during and/or analyzed during the current study are available from the corresponding author on reasonable request.
