# Supplementary material for: Ovary abortion is prevalent in diverse maize inbred lines and is under genetic control
Source: Sci Rep. 2018 Aug 29;8:13032. doi: 10.1038/s41598-018-31216-9 (PMC6115450; doi:10.1038/s41598-018-31216-9)
Supplement: Supplementary file 1 — Dataset 1 [file 41598_2018_31216_MOESM1_ESM.docx]

Ovary abortion is prevalent in diverse maize inbred lines and is under genetic control.

*Authors:* Jeffery L Gustin^a*^, Susan K Boehlein^a^, Janine R Shaw^a^, Weschester Junior^b^, A. Mark Settles^a^, Ashley Webster^c^, William F Tracy^c^, L. Curtis Hannah^a^

Correspondence to: [jgustin@ufl.edu](mailto:jgustin@ufl.edu)

Supplementary Table S1. Kernel abortion in 26 NAM parental inbred lines.

Observed inbred ears (n), genotypic means (µ), and standard deviations (σ) in the 2015 Florida spring and fall nurseries.

| **Season** | **Genotype** | **n** | **Silk (µ)** | **Kernel (µ)** | **Silk (σ)** | **Kernel (σ)** | **Kernel**  **Abortion (%)** | **Kernel**  **Abortion**  **sd (%)** |
| --- | --- | --- | --- | --- | --- | --- | --- | --- |
| spring | B73 | 9 | 512 | 383 | 85.2 | 66.7 | 25.2 | 12.9 |
| fall | B73 | 21 | 473 | 365 | 54.5 | 41.2 | 22.8 | 8.9 |
| spring | B97 | 4 | 432 | 331 | 43.9 | 51.7 | 23.2 | 12.4 |
| fall | B97 | 5 | 379 | 289 | 27.8 | 44.5 | 23.7 | 12.8 |
| spring | CML052 | 4 | 318 | 229 | 20.0 | 36.6 | 28.2 | 8.6 |
| fall | CML052 | 5 | 307 | 208 | 28.5 | 25.9 | 31.4 | 12.8 |
| spring | CML069 | 5 | 300 | 171 | 30.0 | 77.3 | 43.1 | 23.8 |
| fall | CML069 | 6 | 261 | 191 | 39.1 | 38.0 | 26.7 | 5.3 |
| spring | CML103 | 3 | 424 | 328 | 49.6 | 19.7 | 22.7 | 5.0 |
| fall | CML103 | 7 | 336 | 254 | 85.8 | 86.5 | 24.4 | 18.4 |
| spring | CML228 | 16 | 497 | 230 | 54.6 | 54.8 | 53.4 | 10.7 |
| fall | CML228 | 12 | 462 | 235 | 41.4 | 45.8 | 49.2 | 9.5 |
| spring | CML247 | 1 | 420 | 359 | - | - | 14.5 | - |
| fall | CML247 | 2 | 302 | 254 | 8.6 | 12.7 | 15.8 | 6.6 |
| spring | CML277 | 4 | 214 | 127 | 32.8 | 7.4 | 40.6 | 7.6 |
| fall | CML277 | 2 | 275 | 165 | 42.5 | 41.0 | 40.0 | 5.7 |
| spring | CML333 | 2 | 368 | 235 | 63.3 | 79.2 | 36.1 | 8.2 |
| fall | CML333 | 2 | 291 | 260 | 33.8 | 19.8 | 10.7 | 3.6 |
| spring | Hp301 | 5 | 418 | 295 | 19.0 | 62.7 | 29.3 | 13.3 |
| fall | Hp301 | 3 | 418 | 294 | 40.9 | 27.5 | 29.5 | 1.8 |
| spring | Il14H | 5 | 316 | 231 | 81.0 | 48.2 | 27.0 | 7.3 |
| fall | Il14H | 5 | 295 | 222 | 40.4 | 13.7 | 24.8 | 9.6 |
| spring | Ki03 | 5 | 266 | 179 | 33.7 | 67.4 | 32.8 | 30.8 |
| fall | Ki03 | 7 | 335 | 162 | 54.5 | 24.3 | 51.6 | 6.8 |
| spring | Ki11 | 3 | 303 | 223 | 21.2 | 26.1 | 26.4 | 6.5 |
| fall | Ki11 | 5 | 339 | 220 | 91.6 | 40.0 | 35.1 | 8.1 |
| spring | Ky21 | 17 | 428 | 272 | 93.5 | 95.0 | 36.1 | 19.6 |
| fall | Ky21 | 6 | 386 | 291 | 74.6 | 48.9 | 24.5 | 7.9 |
| spring | M037W | 3 | 448 | 293 | 92.1 | 132.9 | 34.7 | 23.9 |
| fall | M037W | 3 | 430 | 293 | 16.5 | 24.8 | 31.8 | 6.2 |
| spring | M162W | 10 | 533 | 294 | 39.4 | 43.9 | 44.8 | 9.7 |
| fall | M162W | 4 | 519 | 284 | 40.3 | 19.6 | 45.2 | 4.8 |
| spring | Mo17 | 5 | 392 | 226 | 67.8 | 33.1 | 42.2 | 15.6 |
| fall | Mo17 | 6 | 267 | 259 | 57.0 | 42.9 | 2.9 | 15.8 |
| spring | Mo18W | 6 | 502 | 334 | 40.6 | 50.8 | 33.4 | 11.7 |
| fall | Mo18W | 4 | 418 | 388 | 48.8 | 38.7 | 7.1 | 2.4 |
| spring | MS71 | 6 | 384 | 301 | 79.4 | 33.4 | 21.6 | 13.2 |
| fall | MS71 | 7 | 331 | 222 | 83.5 | 54.0 | 32.8 | 14.5 |
| spring | NC350 | 4 | 498 | 65 | 40.7 | 30.5 | 87.1 | 7.4 |
| fall | NC350 | 6 | 493 | 173 | 58.3 | 75.6 | 64.9 | 16.9 |
| spring | NC358 | 3 | 365 | 165 | 37.7 | 54.1 | 54.7 | 19.2 |
| fall | NC358 | 4 | 402 | 184 | 124.3 | 36.2 | 54.4 | 10.3 |
| spring | Oh43 | 9 | 459 | 392 | 69.4 | 71.3 | 14.5 | 12.0 |
| fall | Oh43 | 6 | 424 | 297 | 42.4 | 48.7 | 30.0 | 15.3 |
| spring | Oh7B | 4 | 667 | 476 | 43.5 | 60.4 | 28.6 | 10.4 |
| fall | Oh7B | 3 | 448 | 281 | 136.9 | 97.9 | 37.3 | 5.9 |
| spring | P39 | 5 | 516 | 428 | 95.3 | 62.8 | 17.2 | 15.7 |
| fall | P39 | 14 | 514 | 349 | 86.1 | 117.1 | 32.2 | 19.1 |
| spring | Tx303 | 4 | 512 | 273 | 61.0 | 46.1 | 46.7 | 4.8 |
| fall | Tx303 | 4 | 454 | 363 | 34.3 | 41.3 | 20.1 | 4.9 |
| spring | Tzi8 | 5 | 545 | 451 | 70.3 | 55.7 | 17.3 | 7.8 |
| fall | Tzi8 | 17 | 381 | 302 | 52.1 | 44.7 | 20.7 | 12.0 |
